# Precision Psychiatry in Treatment-Resistant Schizophrenia: Clinical Application of Pharmacogenetics in Antipsychotic Treatment

**DOI:** 10.1192/j.eurpsy.2026.11768

**Published:** 2026-07-31

**Authors:** A. Rebollo Díez, P. Fernández Perea

**Affiliations:** Psiquiatría, Complejo Universitario de León, León, Spain

## Abstract

**Introduction:**

Schizophrenia is a severe and chronic mental disorder affecting around 1% of the global population, ranking among the leading causes of disability worldwide. Approximately 20–30% of patients develop treatment-resistant schizophrenia (TRS), characterized by insufficient response to at least two antipsychotics administered at adequate doses and durations. TRS is associated with prolonged hospitalizations, greater cognitive impairment, and reduced quality of life. Precision psychiatry offers new strategies to personalize treatments. In particular, pharmacogenetics allows the study of CYP450 variants (CYP2D6, CYP3A4, CYP1A2, CYP2C19), which play a key role in antipsychotic metabolism, influencing efficacy and safety. This approach could optimize treatment response, reduce adverse effects, and improve functional and cognitive outcomes.

**Objectives:**

To evaluate the clinical utility of pharmacogenetic testing in TRS, focusing on its potential to optimize antipsychotic treatment, improve safety and efficacy, and enhance global functioning and cognition.

**Methods:**

A prospective, longitudinal pre–post study is being conducted in patients with TRS recruited from psychiatric services in León (long-stay units, rehabilitation wards, day hospitals, and community facilities). Participants undergo baseline clinical, cognitive, and genetic assessments, including PANSS, CGI, UKU, FAST, MoCA, TMT-A/B, and DSST, alongside CYP450 genotyping. After pharmacogenetic reports are delivered to treating psychiatrists, patients are reassessed at three months to evaluate changes in antipsychotic dosing, polypharmacy, symptoms, side effects, functioning, and cognition.

**Results:**

The study is expected to provide insight into the prevalence of CYP450 variants in this population, their correlation with clinical practice dosing, and the potential benefits of pharmacogenetic-guided prescribing. By integrating genetic information into real-world clinical decision-making, the project seeks to explore whether this strategy can improve outcomes in TRS and optimize healthcare resource utilization.

**Conclusions:**

Pharmacogenetics may become a valuable tool in precision psychiatry, particularly for patients with TRS. Its implementation could guide more rational prescribing, reduce unnecessary polypharmacy, and support functional recovery. The study is currently ongoing, and the data available by the time of the Congress will be presented.

**Disclosure of Interest:**

None Declared

